# Protease inhibitor ASP enhances freezing tolerance by inhibiting protein degradation in kumquat

**DOI:** 10.1093/hr/uhad023

**Published:** 2023-02-16

**Authors:** Hua Yang, Ke-wei Qiao, Jin-jing Teng, Jia-bei Chen, Ying-li Zhong, Li-qun Rao, Xing-yao Xiong, Huang Li

**Affiliations:** College of Bioscience and Biotechnology, Hunan Agricultural University, Changsha 410128, China; Hunan Provincial Key Laboratory for Germplasm Innovation and Crop Utilization, Hunan Agricultural University, Changsha 410128, China; College of Bioscience and Biotechnology, Hunan Agricultural University, Changsha 410128, China; College of Bioscience and Biotechnology, Hunan Agricultural University, Changsha 410128, China; College of Bioscience and Biotechnology, Hunan Agricultural University, Changsha 410128, China; College of Bioscience and Biotechnology, Hunan Agricultural University, Changsha 410128, China; College of Bioscience and Biotechnology, Hunan Agricultural University, Changsha 410128, China; Hunan Provincial Key Laboratory for Germplasm Innovation and Crop Utilization, Hunan Agricultural University, Changsha 410128, China; Agricultural Genomics Institute at Shenzhen, Chinese Academy of Agricultural Sciences, Shenzhen 518000, China; Center for Plant Science Innovation, University of Nebraska-Lincoln, Lincoln, NE 68588, USA

## Abstract

Cold acclimation is a complex biological process leading to the development of freezing tolerance in plants. In this study, we demonstrated that cold-induced expression of protease inhibitor FmASP in a *Citrus*-relative species kumquat [*Fortunella margarita* (Lour.) Swingle] contributes to its freezing tolerance by minimizing protein degradation. Firstly, we found that only cold-acclimated kumquat plants, despite extensive leaf cellular damage during freezing, were able to resume their normal growth upon stress relief. To dissect the impact of cold acclimation on this anti-freezing performance, we conducted protein abundance assays and quantitative proteomic analysis of kumquat leaves subjected to cold acclimation (4°C), freezing treatment (−10°C) and post-freezing recovery (25°C). FmASP (Against Serine Protease) and several non-specific proteases were identified as differentially expressed proteins induced by cold acclimation and associated with stable protein abundance throughout the course of low-temperature treatment. FmASP was further characterized as a robust inhibitor of multiple proteases. In addition, heterogeneous expression of *FmASP* in *Arabidopsis* confirmed its positive role in freezing tolerance. Finally, we proposed a working model of FmASP and illustrated how this extracellular-localized protease inhibitor protects proteins from degradation, thereby maintaining essential cellular function for post-freezing recovery. These findings revealed the important role of protease inhibition in freezing response and provide insights on how this role may help develop new strategies to enhance plant freezing tolerance.

## Introduction

Freezing injury is a recurrent meteorological hazard that mainly affects overwintering crops, fruit trees, and economic forests [1, 2]. High-value horticultural crops are vulnerable to the threat of freezing temperatures, which have become more frequent in recent years due to climate variability [3, 4]. To cope with and survive freezing temperatures, plants have evolved a series of cold responsive mechanisms which can be placed into two general categories: tolerance and avoidance [5]. Freezing tolerance, defined as the ability of plants to survive extracellular freezing, is accomplished by loss of cellular water to extracellular ice, and the concomitant decease of the freezing point in the cytoplasm. It involves a cascade of transcriptomic and biochemical changes that are frequently present in species found in locations where freezing events are severe and of long duration [5–7]. On the other hand, freezing avoidance mainly involves biophysical changes that regulate ice formation by allowing pockets of water to remain undercooled to very low temperatures, so that the supercooled cells are not exposed to the dehydrative effects and remain in a metastable condition. Avoidance by deep supercooling is widely used by temperate tree species, such as in their xylem parenchyma cells and floral buds, where the intensity of freezing events is moderate and of short duration [8–10]. Strictly speaking, freezing tolerance and avoidance are not mutually exclusive, as in both scenarios plants are trying to avoid or minimize cellular damage and hence improve their hardiness to freezing stress [5, 11].

In the past two decades, with the advent of modern molecular biology and genetic resources, the cold signaling pathway and the underlying regulatory mechanisms of plant cold response have been extensively studied in model species *Arabidopsis* and staple crops [12–16]. One such mechanism is cold acclimation, an adaptive process by which plants develop enhanced freezing tolerance upon exposure to a period of low but non-freezing temperatures. Substantial progress has been made in the characterization of this inducible process at physiological, biochemical and molecular levels [6, 13, 17]. In addition, the temporal pattern of cold stress has been recently recognized as an important factor that affects plant cold response and adaptation [18–20]. Despite these advances, our understanding of cold acclimation in overwintering fruit crops, particularly its role in post-freezing recovery, remains unclear.

Proteomic analysis has been widely employed to reveal the molecular dynamics of plant stress response [21–23]. Alterations in protein metabolism, including protein biosynthesis and protein degradation during acclimation, have been identified as key features that contribute to plant cold tolerance [24]. Proteases and corresponding protease inhibitors (PIs) are direct mediators of protein abundance and quality control in plant cells. They operate in an antagonistic fashion to maintain proteolytic homeostasis at almost all stages and processes of plant life [25]. Although several types of proteases and PIs have been reported to participate in plant immunity and abiotic stress responses [26–28], their function in plant cold or freezing tolerance is largely unknown.

Kumquat [*Fortunella margarita* (Lour.) Swingle] is a subtropical shrub widely cultivated for its rich nutrients and bioactive compounds. It is a close relative to *Citrus* and commonly used in citrus fruit research [29, 30] and breeding for enhanced disease resistance and cold tolerance [31, 32]. Compared to deciduous species including the other cold-hardy *Citrus* rootstock trifoliate orange [*Poncirus trifoliate* (L.) Raf], kumquat is evergreen and more closely related to major commercial *Citrus* species, such as mandarins [*Citrus. reticulata* Blanco], pummelos [*Citrus maxima* Merrill] and citrons [*Citrus. medica* L.] [33, 34]. While the hardiness of trifoliate orange has been well characterized at the molecular and genetic level in recent years [35–37], the mechanism of cold response and tolerance in kumquat is poorly understood with scarce physiological studies available in the literature [38, 39]. Therefore, molecular characterization of cold acclimation and its impact on freezing tolerance in kumquat could close that knowledge gap and provide more transferable information for citrus improvement.

Here, to investigate how cold acclimation affects the anti-freezing capability of kumquat observed in our physiological assays, we carried out a global proteomic analysis of proteins that were responsive to low temperature treatments with a focus on those specifically induced by cold acclimation. Furthermore, we demonstrated how a cold-induced protease inhibitor in kumquat, designated as FmASP, contributes to plant freezing response and tolerance.

## Results

### Cold acclimation enhances the freezing tolerance of kumquat

**Figure 1:**
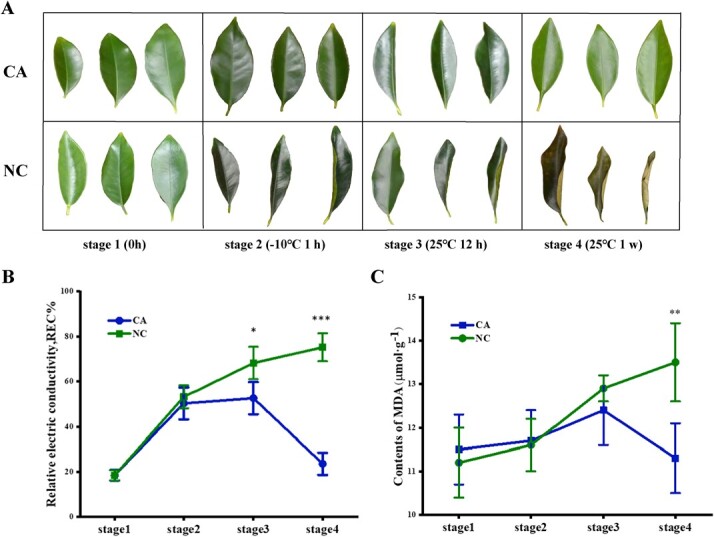
Cold acclimation confers freezing tolerance in kumquat plants. A, Phenotypic changes of cold-acclimated (CA) kumquat leaves subjected to freezing treatment in comparison to the non-acclimated control (NC). Stage 1, the end of cold acclimation; Stage 2, 1 h after −10°C freezing treatment; Stage 3, 12 h recovery at 25°C; Stage 4, one-week recovery at 25°C. B, Measurement of relative electrical conductivity (REC) of kumquat leaves from the CA group and the NC at those four designated stages. C, Measurement of malondialdehyde (MDA) content of kumquat leaves from the CA group and the NC at each stage. Error bars indicate ± SE (n = 3). Asterisks indicate significant differences between the CA plants and the NC at the same stage (^*^ P < 0.05, ^**^ P < 0.01, ^***^ P < 0.001, Student’s *t*-test).

We performed a freezing treatment experiment on kumquat by comparing plants that were cold-acclimated (CA) at 4°C for two weeks with non-acclimated control (NC) plants (stage 1). Without cold acclimation, kumquat leaves started to show frost damage around 1 h at −10°C (stage 2) and suffered severe deformation and dehydration after 12 h recovery at 25°C (stage 3). Permanent damage characterized by crisp texture and dark coloration was observed after recovery at 25°C for one week (stage 4). In comparison, leaves from cold-acclimated kumquat plants exhibited minor changes in coloration during the treatment and restored their color and shape in a week (Figure 1A). Similarly, the whole plants from the CA group resumed normal phenotype while the NC group suffered severe irreversible freezing damage and gradually wilted during the recovery period (Figure S1).

Membranes play a key role in the cold sensing and freezing tolerance in plant cell [40, 41]. Electrolyte leakage and malondialdehyde (MDA) content are widely used as physiological indicators for membrane damage induced by various stresses [42, 43]. In this study, relative electric conductivity (REC) of kumquat leaves from the CA and NC group was quickly increased from 18.4% to 50.3% and 18.5% to 53.2% respectively at stage 2. REC of the CA and NC group continued to increase and reached 52.6% and 68.2% at stage 3. After recovery for one week (stage 4), REC of the control was 75.2% while REC of the CA group was decreased to 20%, close to the pre-treatment level (stage 1) (Figure 1B). Likewise, MDA content showed no significant difference between the CA group and the NC at stage 1 and 2. At the period of recovery, MDA content of the NC group exhibited a sharp increase at both stages (stage 3 and 4) while that of the CA group was increased first but then decreased to a level similar to stage 1 (Figure 1C). Taken together, our observation of phenotypic recovery in the CA group (Figure 1A) and the rebound of REC levels and MDA content in CA plants (Figure 1B, 1C) indicated that the extensive cellular damage caused by freezing were mitigated by cold acclimation in kumquat.

### Cold acclimation maintains protein stability in kumquat leaves during freezing stress

**Figure 2:**
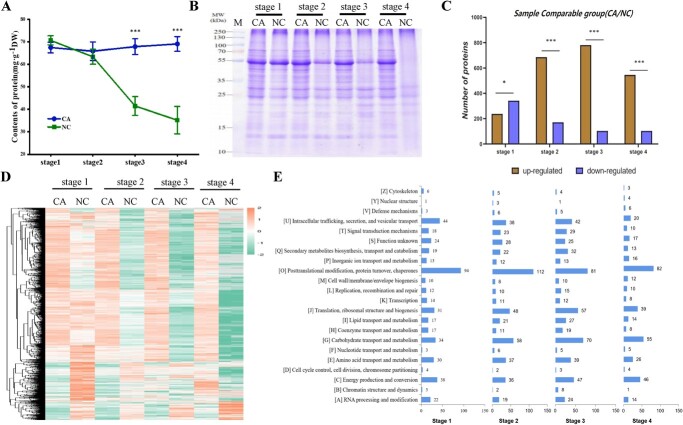
Cold acclimation contributes to the maintenance of protein abundance in kumquat leaves during freezing. A, Measurement of protein content in kumquat leaves from the CA group and the NC at four designated stages (Stage 1, the end of cold acclimation; Stage 2, 1 h after −10°C freezing treatment; Stage 3, 12 h recovery at 25°C; Stage 4, one-week recovery at 25°C). Error bars indicate ± SE (n = 3). Asterisks indicate significant differences between the CA plants and the NC at the same stage (^***^ P < 0.001). B, SDS-PAGE analysis of total protein in kumquat leaves from the CA group and the NC at four designated stages. C, Number of up-regulated and down-regulated proteins in the CA group compared to the NC. Asterisks indicate significant differences between the number of up-regulated proteins and that of down-regulated proteins in the CA group as compared to the NC at each stage (^*^P < 0.05; ^***^ P < 0.001). D, Proteomic heat map of differentially expressed proteins (DEPs) between the CA group and the NC at four designated stages. E, Functional classification of DEPs identified by proteomic analysis. Number of proteins in each category is shown on the right of each bar.

Freezing stress is known to trigger a series of biochemical and physiological changes in plants at the protein level, including the alteration of protein abundance and the production of cold-responsive proteins [24, 44]. Using protein content assay, it was found that while the NC group exhibited significant losses of total protein at stage 3 and 4, the CA group maintained its protein content at a similar level throughout the treatment (Figure 2A). A similar pattern of protein abundance change among the stages of the CA and NC group was observed based on the gel band intensity in SDS-PAGE analysis (Figure 2B).

To identify differentially expressed proteins (DEPs) during the freezing treatment experiment, we performed a global proteomic analysis on proteins extracted from kumquat leaf samples at all aforementioned stages. As a result, 3799 redundant proteins were confidently detected and quantified (Table S1, Figure S2). Using the same confidence level (fold change >1.5), we detected 580 DEPs that were induced by cold acclimation between the CA and NC group. It was found that the 1 h freezing resulted in a significant increase on the number of DEPs, especially the number of up-regulated proteins between the CA and NC at late stages (Figure 2C, Table S2). This distinct pattern of DEPs and the protein abundance change on SDS-PAGE gel indicated that cold acclimation indeed impacted the freezing response of kumquat leaves at the proteomic level, which implies that the proportional change of upregulated protein abundance between CA and NC group could result from either increased *de novo* protein synthesis in CA, increased protein degradation in NC, or a combination of both. Thus, we next conducted a clustering analysis to assess individual protein changes based on normalized abundance at the four stages (Figure 2D). In general, the expression of a large number of clustered proteins from the NC group was reduced by freezing (stage 2) and continued the decreasing trend in the recovery phase (stage 3 and 4). In contrast, the expression of most proteins from the CA group remained constant in later stages (stage 3 and 4) after freezing treatment. More interestingly, some proteins downregulated by cold acclimation (stage 1) without any significant change when exposed to freezing stress eventually restored their abundance during recovery (stage 4). Functional classification indicated that the DEPs were mostly involved in four categories: 1) posttranslational modification, protein turnover, chaperons; 2) intracellular trafficking, secretion and vesicular transport; 3) energy production and conversion; and 4) metabolic activities (Figure 2E). These results suggested that most of the acclimation-responsive proteins either maintained or gradually retained their abundance while the non-acclimated group experienced significant fluctuation and loss of proteins during the phases of freezing treatment and recovery. Therefore, we concluded that cold acclimation is a critical process that confers kumquat’s freezing tolerance by stabilizing protein abundance.

### Cold acclimation affects specific expression of proteases and protease inhibitors

To further dissect the role of cold acclimation in freezing tolerance at the proteomic level, we focused on the identified DEPs between the CA and NC group at stage 1. Differential protein abundance can be the result of several processes including changes in *de novo* protein biosynthesis, protein modification and protein degradation. Cold-responsive proteins and their post-translational modifications, including protein phosphorylation, ubiquitination and SUMOylation have been reported to be key regulators of plant cold signaling and freezing tolerance [14, 16, 45]. Not surprisingly, a number of these well-characterized proteins were identified among the DEPs at the acclimation and freezing stages in our proteomic analysis (Table S3). However, few in-depth studies have been conducted on the topic of protein degradation, a crucial process of plant stress responses in which proteases and protease inhibitors are major players [46–48]. In this study, we identified a total of 31 cold-induced proteases, including the specific ATP-dependent caseinolytic proteases (Clp), cysteine proteases, and non-specific ones such as serine proteases and aspartyl proteases (Table S4). Interestingly, the majority of non-specific proteases, predictively localized to the chloroplast, were found to be down-regulated by cold acclimation. Moreover, we identified two differentially expressed protease inhibitors, ASP and KTI2 (Table S4). Notably, the upregulated ASP is homologous to AtKTI5 (*At1g17860.1*), a member of the Kunitz trypsin inhibitor (KTI) family. KTI protease inhibitors are known to be involved in plant immunity and stress resistance by modulating protease activity [26, 46]. Taken together, we hypothesized that the cold-induced ASP could play a key role in modulating freezing tolerance at the proteomic level in kumquat.

### FmASP acts as a major protease inhibitor in kumquat freezing response

**Figure 3:**
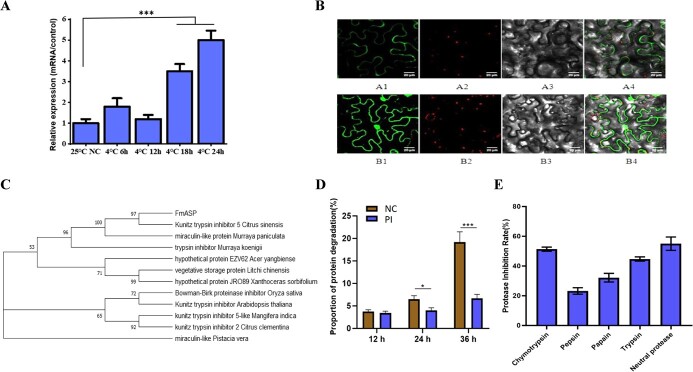
FmASP is a cold-inducible extracellular protease inhibitor in the KTI family. A, Relative gene expression of FmASP in the leaves of kumquat under cold treatment as detected by qRT-PCR. Error bars indicate ± SE (n = 3). Asterisks indicate significant differences between the time points of treatment (^***^ P < 0.001). B, Subcellular localization of FmASP in tobacco cells revealed by confocal microscopy. A1 represents the GFP fluorescence signal of the target gene; B1 indicates that the GFP fluorescence signal from an empty vector as the control; A2 and B2 indicate the chloroplast fluorescence signal; A3 and B3 represent the open bright field; A4 and B4 are the merged images of bright field, GFP and the chloroplast fluorescence. C, Phylogenetic analysis of FmASP. Numbers above branches indicate bootstrap values. D, *In vitro* protein degradation assay using FmASP as the protease inhibitor (PI). The inhibitory activity of PI was assessed as the percentage of reduction in protein concentration relative to that of the control (NC). Error bars indicate ± SE (n = 3). Asterisks indicate significant differences between two groups at each time point of incubation (^*^P < 0.05, ^***^ P < 0.001, Student’s *t*-test). E, *In vitro* inhibitory assay on common proteases using 10 μg/ml FmASP protein or equal volume of PBS buffer. Ratio of inhibition rates on proteases (20 μg/ml) with versus without FmASP was calculated. Error bars indicate ± SE (n = 3).

To explore how FmASP functions as a protease inhibitor in the freezing response of kumquat, we studied its temporal expression, subcellular localization and inhibitory activity. Consistent with the results of proteomic analysis, FmASP was up-regulated by cold treatment (Figure 3A) and the FmASP protein was predicted to be located in the extracellular space (Figure 3B), as revealed by qRT-PCR and the transient expression experiment, respectively. In addition, phylogenetic analysis revealed that FmASP was homologous to proteins characterized as Kunitz trypsin inhibitors in multiple species including its closest *Citrus* relative (Figure 3C). To test the inhibitory function of FmASP, we first expressed and purified the recombinant His-FmASP protein (Figure S3), and then conducted *in vitro* protein degradation and protease activity assays. It was found that the addition of 10 μg/ml active FmASP protein significantly reduced the degradation rate of soluble protein extract (initial concentration 200 μg/ml) from kumquat leaves (Figure 3D). Specifically, its inhibitory effect can last up to 36 h to keep protein degradation at a much lower level compared to the control. Moreover, FmASP reduced the total proteolytic activity of common proteases and neutral protease as suggested in the inhibitory assay (Figure 3E). Among them, chymotrypsin and trypsin were the most effectively inhibited by FmASP, reflecting its role as a Kunitz trypsin inhibitor.

### Heterogeneous expression of *FmASP* enhances freezing tolerance in *Arabidopsis*

**Figure 4:**
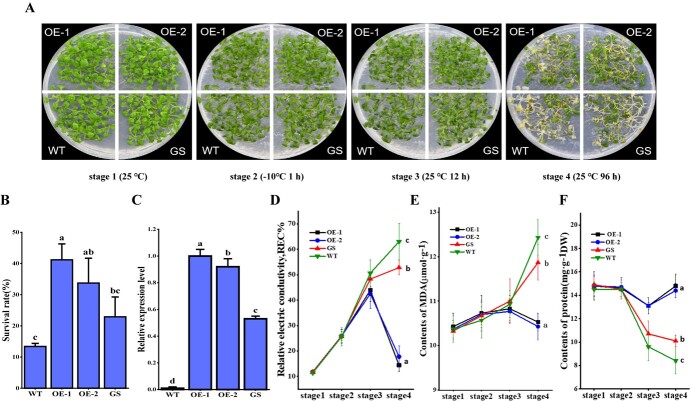
Overexpression of *FmASP* confers freezing tolerance in transgenic *Arabidopsis* plants. A, Plant phenotypes of the transgenic *Arabidopsis* lines and WT before and after the cold treatment. OE-1, overexpression line 1; OE-2, overexpression line 2; GS, gene-silencing line; WT, wild type. Stage 1: three-week-old seedlings grown at 25°C; Stage 2: 1 h after −10°C freezing treatment; Stage 3: 12 h recovery at 25°C; Stage 4: 96 h recovery at 25°C. B, Survival rates of transgenic plants compared to the WT at stage 4. C, Relative gene expression of *FmASP* in the transgenic plants and WT as detected by qRT-PCR. Normalized Ct values were obtained from three independent biological replicates of each line including the WT and calculated by the 2^-ΔΔCt^ method. D, Measurement of relative electrical conductivity (REC) in the transgenic plants and WT at all four stages. E, Measurement of the content of malondialdehyde (MDA) in the transgenic plants and WT at all four stages. F, Measurement of the protein content in the transgenic plants and WT at all designated stages. All data in B-F are shown as means of three replicates ± SE. Different letters on the top of each bar or beside each time point denote statistically significant differences among the lines at P < 0.05 level.

To further confirm the protease inhibitory function of FmASP in freezing stress response, we generated stable transgenic *Arabidopsis* plants that overexpressed the *FmASP* gene and subjected them to freezing tolerance assay. We also generated independent *FmASP*-silencing *Arabidopsis* plants by RNAi in the background of the overexpressed line for comparison. The results showed that compared to wild type, overexpression of *FmASP* significantly enhanced freezing tolerance as indicated by higher survival rates at the recovery stage (Figure 4A-C). Additionally, measurements of electrical conductivity and MDA content further indicated that the membrane damage incurred by freezing was restored in *FmAS*P-overexpressing plants (Figure 4D, 4E). An assay of total protein content also revealed that *FmASP*-overexpression lines maintained their protein content at a more constant level after freezing, compared to the wild type (Figure 4F). On the other hand, the *FmASP*-silencing plants exhibited an intermediate survival rate and substantial cellular damage similar to that of the wild type (Figure 4A-F). These results implied that *FmASP* can enhance freezing tolerance in *Arabidopsis* by maintaining overall protein stability and largely restoring cellular injury to a pre-freezing level, enabling plants to resume growth.

## Discussion

Plant cold acclimation is very complex, involving a series of inducible mechanisms to protect cells from freezing injury [6, 17]. Among those cryoprotective mechanisms, protein stabilization plays a crucial role in maintaining the structure and function of delicate cell membranes during freezing stress [12, 49]. Plant proteases are ubiquitous enzymes that play an essential role in modulating protein quality and homeostasis to acclimatize to environmental stresses [47, 50]. Several types of proteases have been characterized to be associated with plant responses to drought [51, 52], salinity [53–55] and biotic stresses [48, 56]. In this study, we investigated the temporal changes of protein abundance in kumquat plants under a regime of cold acclimation, freezing exposure, and post-freezing recovery. It was found that cold-acclimated plants had better control over protein content stability than their non-acclimated counterparts during the whole treatment. Prompted by this observation, we used a comparative proteomic approach to quantify changes that might lead to protein homeostasis and overall freezing tolerance in kumquats. We identified 31 proteases that were significantly induced by cold acclimation, which was the key step in determining whether a kumquat plant can recover from severe freezing injury indicated by membrane ion leakage and oxidative damage. These differentially expressed proteases are distributed across different cellular compartments, especially on the plastid membranes (Table S4). They mainly include ATP-dependent Clp (serine-type) proteases and cysteine proteases, two protease families with potential implications in plant stress responses [53, 54]. Specifically, Clp proteases are a prominent protease family located in the chloroplast that degrades numerous stromal proteins [57]. Cysteine proteases are the best characterized protein proteases involved in many processes, particularly those associated with storage protein degradation and programmed cell death [58, 59]. Taken together, we concluded that the induction of the Clp, cysteine proteases and other non-specific proteases during cold acclimation could be one of the main factors contributing to freezing tolerance in kumquat by increasing protein stability.

Moreover, we identified a cold-induced protease inhibitor FmASP in proteomic study and characterized its function. Protease inhibitors are multifunctional proteins implicated in the control of endogenous proteolysis under biotic and abiotic stress conditions [26, 46]. Constitutive expression of protease inhibitors has been shown to modulate the activity of endogenous proteases and confer multiple stress tolerance in transgenic plants [60–62]. However, the specific involvement and mechanism of protease inhibitors in plant cold response are not well understood. In agreement with the positive role of protease inhibitors in plant immune and stress responses, our study found that FmASP, a member of the Kunitz trypsin inhibitor (KTI) family, confers freezing tolerance in kumquat and *Arabidopsis* plants. *In vitro* protein degradation and enzyme activity assays using purified FmASP protein confirmed its role as a protease inhibitor. Additionally, we observed a gradually increased expression pattern of *FmASP* at 4°C and that the inhibitory activity of FmASP can last for at least 36 h, reflecting its role as a plant defensive chemical in natural conditions. Overexpression of *FmASP* in *Arabidopsis* enhanced freezing tolerance, whereas *FmASP*-silenced transgenic lines were more susceptible to freezing damage. Notably, the degree of cellular damage, as indicated by ion leakage, MDA quantification, and soluble protein content, largely corroborated the FmASP expression level of transgenic plants. The robust and tunable nature of FmASP as a protease inhibitor makes it a promising candidate for future plant engineering and trait development.

Subcellular localization assays revealed that FmASP is an extracellular protease inhibitor. One question that arises is how FmASP enters the cells from the apoplast during cold acclimation, as this process appears to be induced yet highly regulated. There is evidence that protease activity can be controlled by physically separating the enzyme from its substrates or remobilizing the enzyme to its substrates in specific cellular compartments [57]. Several plant proteases and protease inhibitors have been found to enter the cells primarily through endocytosis, a sophisticated mechanism which allows for the avoidance of unwanted proteolytic enzymes in the cytosol that could degrade essential constituents [63–65]. Our proteomic analysis revealed that multiple proteins from the endocytic pathway, such as the previously characterized RAB GTPase family [66, 67], were upregulated under cold conditions (Figure S4, Table S5). Further investigation of the activation mechanism of endocytosis and the mechanistic trafficking of FmASP will help to better understand these understudied areas of plant cold acclimation.

**Figure 5:**
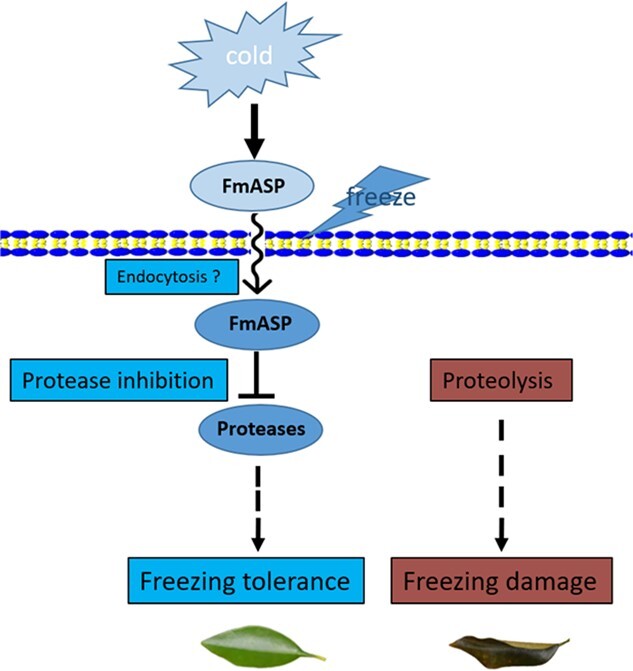
A proposed working model of FmASP in cold acclimation-induced freezing tolerance. In contrast to the dramatic proteolysis and cellular damage caused by freezing in non-acclimated plants, cold-acclimated plants have a different response, which could be described as follows. 1) cold acclimation induces the expression of extracellular FmASP. 2) These FmASP proteins accumulate in the extracellular space may enter the cell through endocytosis. 3) During freezing, the FmASP proteins are released to the cytoplasm and in close contact with cytosolic proteases; 4) FmASP acts as a protease inhibitor, effectively protecting vital proteins that are necessary for freezing response and tolerance from degradation.

Based on the results of our study and a review of current literature [59, 63, 64, 68], we propose a new strategy for enhancing plant resilience through the involvement of proteases and protease inhibitors that confer freezing tolerance in kumquat (Figure 5). In contrast with the dramatic cellular or vacuolar proteolysis caused by freezing in non-acclimated plants, cold acclimation triggers the downregulation of several non-specific proteases from different cellular compartments and the upregulation of extracellular-localized FmASP. FmASP could enter the cells via the process of endocytosis. At the onset of freezing temperatures, ice formation causes severe damage to cellular membranes, including those of endocytic vesicles. Then, FmASP is released into the cytoplasm and comes into close contact with cytosolic proteases. Along with other proteins induced by cold temperatures, its function and enzymatic activity are halted, but mostly preserved at freezing temperatures. Upon recovery at more favorable temperatures, FmASP acts to inhibit various proteases, thereby largely preventing the protein degradation that occurred in non-acclimated plants. As a result, most functional proteins, including those cold-responsive and cell-damage-repair proteins, are able to avoid degradation and resume their function to cope with freezing stress, thereby enhancing freezing tolerance. This proposed working model of protease inhibitor will be of great importance for stimulating further studies of cold acclimation on plant freezing tolerance and provide a new perspective to manipulate the responsive mechanisms of environmental stresses in temperate fruit tree species.

## Materials and methods

### Phenotypic observation of kumquat plants under freezing stress

Six-month-old kumquat [*F. margarita* (Lour.) Swingle] seedlings from the National Center for Citrus Improvement (Changsha, China) were transplanted to pots with the compost soil (potting soil, turfy soil, and vermiculite 1:1:1) and grown for about 1.5 years in a greenhouse, with ambient temperature between 25 and 30°C. We selected twenty well-established plants of similar size for the following treatment regime. Ten plants were transferred to a climate-controlled growth chamber for cold acclimation (CA) group at 4°C while the other ten plants were grown in parallel at 25°C as the non-acclimated control (NC) group (stage 1). After two weeks, all plants were placed in a freezing chamber (−10°C) for 1 h (stage 2) and then transferred to the growth chamber for recovery. The plants recovered in the chamber at 25°C for 12 h and for one week were recorded as stage 3 and 4, respectively. Three independent freezing tolerance assays were performed as replicates. Representative leaves and whole plants from each stage were selected for photographing. The sample leaves were collected at those four designated stages, flash frozen in liquid nitrogen for subsequent analysis.

### Measurement of physiological indicators in freezing-stressed leaf tissue

Relative electric conductivity (REC) was measured using a Mettler Toledo FE30 conductivity meter. Kumquat leaf samples were placed into 20 mL tubes containing 10 mL deionized water. The solution was vacuumed for 30 min and the conductance of water was measured as S0. After shaken at room temperature for 1 h, the conductance of water was measured as S1. Then samples were boiled in boiling water for 30 min and shaken at room temperature for 1 h with the conductance measured as S2. The value of (S1-S0)/(S2-S0) was calculated as the REC.

Malondialdehyde (MDA) content was determined by spectrophotometry following the protocol [69, 70] with slight modification. Briefly, fresh kumquat leaves (0.5 g) were ground with a mortar and pestle and homogenized in 1 mL of 10% trichloroacetic acid (W/V). The homogenate was transferred to a centrifuge tube, washed with 1 mL 10% trichloroacetic acid, and centrifuged for 10 min at 10000 g. Next, 1 mL of supernatant was mixed with 1 mL of 0.6% thiobarbituric acid and incubated in boiling water for 30 min. After brief cooling on ice, the solution was centrifuged at 3000 g for 10 min, and the absorbance of the supernatant was measured at 450, 532 and 600 nm respectively. The content was calculated as MDA (μmol·g^−1^) = 6.45 × (A532 − A600) − 0.56 × A450.

The soluble protein content of kumquat leaves from each designated stage was measured by the Coomassie brilliant blue method. All measurements were conducted in triplicates.

### Protein preparation, digestion and LC–MS/MS analysis

Approximately 500 mg leaves sampled from the CA or NC group at each stage was ground thoroughly in liquid nitrogen and extracted with 5 mL 1% SDS lysis buffer containing 10 mM dithiothreitol and 1% protease inhibitor. An equal volume of Tris-saturated phenol (pH 8.0) was added before the mixture was vortexed for 5 min. After centrifugation (4°C, 10 min, 5500 g), the upper phenol phase was transferred to a new centrifuge tube. Proteins were precipitated by adding five volumes of ammonium sulfate-saturated methanol and incubated at −20°C overnight. The resulting precipitates were washed with cold methanol, followed by three washes with acetone [71]. Protein concentration was determined using a bicinchoninic acid (BCA) protein assay kit (Beyotime, China). For digestion, equal amount of protein from each sample was adjusted to the same volume using lysis buffer and then re-precipitated with 20% TCA at 4°C for 2 h. After washing twice with ice-cold acetone, the precipitate was diluted in 200 mM tetraethylammonium bromide (TEAB, pH 8.0). Finally, trypsin was added for digestion (w/w for enzyme: sample = 1: 50) at 37°C for 16 h. The resulting peptide mixture was reduced with 5 mM dithiothreitol (DTT) for 30 min at 56°C and alkylated with 11 mM iodoacetamide for 15 min at room temperature in darkness.

Proteomic data acquisition was performed on an EASY-nLC 1200 ultra-performance liquid chromatography system connected to an Orbitrap Exploris 480 mass spectrometer (Thermo Scientific, San Jose, CA). For LC–MS/MS proteomic data analysis, we used Maxquant (v1.6.15.0) and the search database (*Citrus_japonica*_76966_TX_20210429.fasta) to process raw MS files. Search results were filtered at 1% false discovery rate (FDR) and peptide confidence level was set as at least one unique peptide per protein for protein identification. The up- or down-regulated proteins with relative quantification *p*-values <0.05 and 1.5 fold-change ratios were selected as differentially expressed proteins. Euclidean distance and hierarchical cluster were used to cluster differentially expressed proteins. Functional annotation and the analysis of annotation data were performed using Blast2GO (https://www.blast2go.com/). KEGG database (http://www.genome.jp/kegg/) and Clusters of Orthologous Groups of proteins (COGs) (http://www.ncbi.nlm.nih.gov/COG/) were used for protein identification, classification, and clustering.

### Subcellular localization, heterologous expression and purification of FmASP

Full-length cDNA of *FmASP* fused with a C-terminal green fluorescent protein (GFP) was cloned into vector pCAMBIA2300. The constructed plasmids were transferred into Agrobacterium strain *EHA105* and then transformed into tobacco (*Nicotiana benthamiana*) leaves for transient expression. Subcellular localization of GFP fusion proteins was detected and captured by a laser confocal microscope (FV1000-IX81, Olympus, Japan).

Recombinant plasmid pBRT7–7806 and the control plasmid pBRT7 were synthesized and transformed into *Escherichia coli* BL21 (DE3) to express FmASP protein. Target protein was recovered and purified with a His-Trap affinity column (QIAGEN) according to manufacturers’ protocols. His-FmASP protein was detected by immunoblotting with a mouse anti-His antibody (GenScript, A00186). The immunoblotting band signals were visualized by enhanced chemiluminescence (ECL) detection system (Tanon, Shanghai, China) and images were obtained using a cooled charge-coupled device (CCD) camera (Tanon-4100).

### Protease inhibition assays

Kumquat leaves were pulverized in liquid nitrogen using a pestle and mortar, weighed and incubated on ice with 10 mL PBS buffer (pH 6.5) for 30 min with occasional mixing. The mixture was centrifuged at 20000 g at 4°C for 30 min and the resulting supernatant was centrifuged two more times. 0.9 mL of the supernatant was placed in a test tube with the addition of 1 μl purified FmASP protein. Total protein content in the solution after incubating at 25°C for 0, 12, 24 and 36 h was measured using the BCA protein assay kit (Beyotime, China). Protease inhibition was assessed as the percentage of reduction in protein concentration relative to that of the control. Control for each set was made by adding equal volume of heat-denatured FmASP. All assays were made with three different pools and in triplicate.

The inhibitory activity of FmASP was measured in PBS buffer containing 20 μg/ml protease cocktail (chymotrypain, pepsin, papain, trypsin and neutral protease) with 200 μg/ml S-7388 substrate (Sigma-Aldrich); final volume was 100 μL. Prior to substrate addition, proteases were incubated for 30 min at 37°C with either 10 μg/ml FmASP or equal volume of PBS buffer. The reaction velocity was measured spectrophotometrically at 450 nm for 5 min using a SpectraMax M5e plate reader (Molecular Devices, USA). Experiments were performed in triplicates and velocities were reported as means ± SE.

### Plasmid construction and generation of transgenic plants

Full-length ASP cDNA obtained from *Fortunella margarita* was amplified by RT-PCR using primers with compatible enzyme digestion sites. The *FmASP* amplicon was confirmed by sequencing and then cloned into a p1300M vector under control of the constitutive cauliflower mosaic virus 35S promoter. The 35S::p1300M-ASP construct was introduced into the *Agrobacterium* strain GV3101 and then transferred into wild-type *Arabidopsis* (Col-0) plants by floral-dip transformation. Transgenic lines obtained were first screened by hygromycin and then verified by PCR. T4 homozygous transgenic lines were selected for freezing tolerance assay and downstream analysis.

Gene silence vector pBWA(V)HS-ASP was constructed by assembling inverted repeats of 200–300 bp fragments of *FmASP* CDS linked by a 200 bp loop sequence and driven by the CaMV 35S promoter. After verified by DNA sequencing, the vector was transformed into the homozygous FmASP overexpression line of *Arabidopsis* to obtain gene silencing (GS) lines. The information of all primers used in this study was presented in Table S6.

### qRT-PCR assay

Total RNA was extracted from 4-week-old *Arabidopsis* plants with TRIzol reagent (Invitrogen). Reverse transcription and quantitative real-time PCR assay were performed in a Bio-Rad iQ5 real-time system using a Quant One Step qRT-PCR (SYBR Green I) Kit (Tiangen Biotech). Transcript expression levels of *FmASP* were obtained in three biological replicates of each line, normalized to that of the reference gene *ACTIN* and calculated using the 2^-ΔΔCt^ method [72].

### Phenotypic observation, physiological assays of transgenic *Arabidopsis* under freezing stress

Three-week-old seedlings grown on ½ MS medium plates from a climate-controlled growth chamber (25°C; stage 1) were placed into a freezing chamber at −10°C for 1 h (stage 2) and then recovered at 25°C for 12 h (stage 3) and 96 h (stage 4) before counting the survival rate. Seedlings with a non-dehydrated stem and at least three green leaves were recorded as the survivors. The MDA, relative electric conductivity and total protein content assays of transgenic *Arabidopsis* were performed using the same methods as described in kumquat experiments.

## Acknowledgments

The authors would like to thank Drs. Xiangyang Lu (Hunan Agricultural University, China), Zhanguo Xin (USDA-ARS, Lubbock, TX, United States) and Huazhong Shi (Texas Tech University, Lubbock, TX, United States) for giving technical guidance and Drs. Zhanguo Xin and Xinbo Chen (Hunan Agricultural University, China) for revision on earlier versions of this manuscript. We also thank Drs. Jeff Mower and Arvind Dubey (University of Nebraska-Lincoln, United States) for their critical feedback and editing. This research was sponsored by the National Natural Science Foundation of China (No.31200963) and the Key Project of Hunan Provincial Education Department (No.18A091).

## Author contributions

H.Y., X.X. and H.L. conceived and designed the experiments. H.Y., K.Q., J. T., J.C., and Y.Z. set up and carried out the experiments. H.Y., K.Q., J.T., Y.Z., L.R. and H.L. analyzed the data. H.Y., Y.Z. and H.L. wrote the paper. All authors reviewed the manuscript.

## Data availability

All data supporting the findings of this work are included in the article and supplementary files. The plant materials used in this study are available from the corresponding authors upon request.

## Conflict of interest statement

The authors declare that they have no conflict of interest.

## Supplementary Data

Supplementary data is available at *Horticulture Research* online.

## Supplementary Material

Web_Material_uhad023

## References

[1] Ding YL , Shi YT, Yang SH. Molecular regulation of plant responses to environmental temperatures. Mol Plant. 2020;13:544–64. 32068158 10.1016/j.molp.2020.02.004

[2] Rapacz M , Ergon A, Hoglind M et al. Overwintering of herbaceous plants in a changing climate. Still more questions than answers. Plant Sci. 2014;225:34–44. 25017157 10.1016/j.plantsci.2014.05.009

[3] Luedeling E . Climate change impacts on winter chill for temperate fruit and nut production: a review. Sci Hortic. 2012;144:218–29.

[4] Malhotra SK . Horticultural crops and climate change: a review. Indian J Agric Sci. 2017;87:12–22.

[5] Gusta LV , Wisniewski M. Understanding plant cold hardiness: an opinion. Physiol Plant. 2013;147:4–14. 22409670 10.1111/j.1399-3054.2012.01611.x

[6] Thomashow MF . Plant cold acclimation: freezing tolerance genes and regulatory mechanisms. Annu Rev Plant Physiol Plant Mol Biol. 1999;50:571–99. 15012220 10.1146/annurev.arplant.50.1.571

[7] Wisniewski M , Willick IR, Gusta LV. Freeze Tolerance and Avoidance in Plants. In Shabala S (ed.), Plant Stress Physiology. Oxfordshire: CABI, 2017, 279–99.

[8] Wisniewski M , Gusta L, Neuner G. Adaptive mechanisms of freeze avoidance in plants: a brief update. Environ Exp Bot. 2014;99:133–40.

[9] George MF , Burke MJ. Supercooling of tissue water to extreme low-temperature in overwintering plants. Trends Biochem Sci. 1984;9:211–4.

[10] Ashworth EN , Wisniewski ME. Response of fruit tree tissues to freezing temperatures. HortScience. 1991;26:501–4.

[11] Pearce RS . Plant freezing and damage. Ann Bot. 2001;87:417–24.

[12] Chang CYY , Brautigam K, Huner NPA et al. Champions of winter survival: cold acclimation and molecular regulation of cold hardiness in evergreen conifers. New Phytol. 2021;229:675–91. 32869329 10.1111/nph.16904

[13] Chinnusamy V , Zhu JK, Sunkar R. Gene regulation during cold stress acclimation in plants. Methods Mol Biol. 2010;639:39–55. 20387039 10.1007/978-1-60761-702-0_3 PMC3064467

[14] Guo XY , Liu DF, Chong K. Cold signaling in plants: insights into mechanisms and regulation. J Integr Plant Biol. 2018;60:745–56. 30094919 10.1111/jipb.12706

[15] Kidokoro S , Yoneda K, Takasaki H et al. Different cold-signaling pathways function in the responses to rapid and gradual decreases in temperature. Plant Cell. 2017;29:760–74. 28351986 10.1105/tpc.16.00669 PMC5435423

[16] Ding YL , Shi YT, Yang SH. Advances and challenges in uncovering cold tolerance regulatory mechanisms in plants. New Phytol. 2019;222:1690–704. 30664232 10.1111/nph.15696

[17] Xin Z , Browse J. Cold comfort farm: the acclimation of plants to freezing temperatures. Plant Cell Environ. 2000;23:893–902.

[18] Vyse K , Pagter M, Zuther E et al. Deacclimation after cold acclimation-a crucial, but widely neglected part of plant winter survival. J Exp Bot. 2019;70:4595–604. 31087096 10.1093/jxb/erz229 PMC6760304

[19] Herrmann HA , Schwartz JM, Johnson GN. Metabolic acclimation-a key to enhancing photosynthesis in changing environments? J Exp Bot. 2019;70:3043–56. 30997505 10.1093/jxb/erz157

[20] Wang LX , Sadeghnezhad E, Nick P. Upstream of gene expression: what is the role of microtubules in cold signalling? J Exp Bot. 2020;71:36–48. 31560041 10.1093/jxb/erz419

[21] Wang PY , Yao SL, Kosami KI et al. Identification of endogenous small peptides involved in rice immunity through transcriptomics- and proteomics-based screening. Plant Biotechnol J. 2020;18:415–28. 31301098 10.1111/pbi.13208 PMC6953209

[22] Kosova K , Vitamvas P, Urban MO et al. Plant abiotic stress proteomics: the major factors determining alterations in cellular proteome. Front Plant Sci. 2018;9:122. 29472941 10.3389/fpls.2018.00122 PMC5810178

[23] Hossain Z , Nouri MZ, Komatsu S. Plant cell organelle proteomics in response to abiotic stress. J Proteome Res. 2012;11:37–48. 22029473 10.1021/pr200863r

[24] Janmohammadi M , Zolla L, Rinalducci S. Low temperature tolerance in plants: changes at the protein level. Phytochemistry. 2015;117:76–89. 26068669 10.1016/j.phytochem.2015.06.003

[25] Rustgi S , Boex-Fontvieille E, Reinbothe C et al. The complex world of plant protease inhibitors: insights into a Kunitz-type cysteine protease inhibitor of Arabidopsis thaliana. Commun Integr Biol. 2018;11:e1368599. 29497469 10.1080/19420889.2017.1368599 PMC5824933

[26] Brzin J , Kidric M. Proteinases and their inhibitors in plants: role in normal growth and in response to various stress conditions. Biotechnol Genet Eng Rev. 1996;13:421–68.

[27] Shan L , Li CL, Chen F et al. A Bowman-Birk type protease inhibitor is involved in the tolerance to salt stress in wheat. Plant Cell Environ. 2008;31:1128–37. 18433440 10.1111/j.1365-3040.2008.01825.x

[28] Clemente M , Corigliano MG, Pariani SA et al. Plant serine protease inhibitors: biotechnology application in agriculture and molecular farming. Int J Mol Sci. 2019;20:1345. 30884891 10.3390/ijms20061345 PMC6471620

[29] Li XH , Meenu M, Xu BJ. Recent development in bioactive compounds and health benefits of kumquat fruits. Food Rev Int. 2022;3:1–21.

[30] Sadek ES , Makris DP, Kefalas P. Polyphenolic composition and antioxidant characteristics of kumquat (Fortunella margarita) Peel fractions. Plant Food Hum Nutr. 2009;64:297–302. 10.1007/s11130-009-0140-1 19866359

[31] Khalaf A , Moore GA, Jones JB et al. New insights into the resistance of Nagami kumquat to canker disease. Physiol Mol Plant Pathol. 2007;71:240–50.

[32] Grosser JW , Chandler TL. Production of twelve new allotetraploid somatic hybrid citrus breeding parents with emphasis on late maturity and cold-hardiness. J Amer Pomol Soc. 2004;58:21–8.

[33] Barkley NA , Roose ML, Krueger RR et al. Assessing genetic diversity and population structure in a citrus germplasm collection utilizing simple sequence repeat markers (SSRs). Theor Appl Genet. 2006;112:1519–31. 16699791 10.1007/s00122-006-0255-9

[34] Krueger RR , Navarro L. Citrus Germplasm Resources. In Khan IA (ed.), Citrus Genetics, Breeding and Biotechnology. Wallingford, UK: CAB International, 2007, 45–140.

[35] Wang M , Zhang XN, Liu JH. Deep sequencing-based characterization of transcriptome of trifoliate orange (Poncirus trifoliata (L.) Raf.) in response to cold stress. BMC Genomics. 2015;16:555. 26219960 10.1186/s12864-015-1629-7 PMC4518522

[36] Zhang Y , Ming RH, Khan M et al. ERF9 of Poncirus trifoliata (L.) Raf. Undergoes feedback regulation by ethylene and modulates cold tolerance via regulating a glutathione S-transferase U17 gene. Plant Biotechnol J. 2022;20:183–200. 34510677 10.1111/pbi.13705 PMC8710834

[37] Peng Z , Bredeson JV, Wu GHA et al. A chromosome-scale reference genome of trifoliate orange (Poncirus trifoliata) provides insights into disease resistance, cold tolerance and genome evolution in citrus. Plant J. 2020;104:1215–32. 32985030 10.1111/tpj.14993 PMC7756384

[38] Young R , Bell WD. Photosynthesis in detached leaves of cold-hardened citrus Seedings. J Amer Soc Hort Sci. 1974;99:400–3.

[39] Santini J , Giannettini J, Pailly O et al. Comparison of photosynthesis and antioxidant performance of several citrus and Fortunella species (Rutaceae) under natural chilling stress. Trees. 2013;27:71–83.

[40] Moellering ER , Muthan B, Benning C. Freezing tolerance in plants requires lipid remodeling at the outer chloroplast membrane. Science. 2010;330:226–8. 20798281 10.1126/science.1191803

[41] Orvar BL , Sangwan V, Omann F et al. Early steps in cold sensing by plant cells: the role of actin cytoskeleton and membrane fluidity. Plant J. 2000;23:785–94. 10998189 10.1046/j.1365-313x.2000.00845.x

[42] Jin R , Wang YP, Liu RJ et al. Physiological and metabolic changes of purslane (Portulaca oleracea L.) in response to drought, heat, and combined stresses. Front Plant Sci. 2016;6:1123. 26779204 10.3389/fpls.2015.01123 PMC4703826

[43] Tajvar Y , Ghazvini RF, Hamidoghli Y et al. Antioxidant changes of Thomson navel orange (Citrus sinensis) on three rootstocks under low temperature stress. Hortic Environ Biotechnol. 2011;52:576–80.

[44] Shi Y , Ding Y, Yang S. Molecular regulation of CBF signaling in cold acclimation. Trends Plant Sci. 2018;23:623–37. 29735429 10.1016/j.tplants.2018.04.002

[45] Barrero-Gil J , Salinas J. Post-translational regulation of cold acclimation response. Plant Sci. 2013;205-206:48–54. 23498862 10.1016/j.plantsci.2013.01.008

[46] Divekar PA , Rani V, Majumder S et al. Protease inhibitors: an induced plant defense mechanism against herbivores. J Plant Growth Regul. 2022;1–17.35431419

[47] Sharma P , Gayen D. Plant protease as regulator and signaling molecule for enhancing environmental stress-tolerance. Plant Cell Rep. 2021;40:2081–95. 34173047 10.1007/s00299-021-02739-9

[48] Thomas EL , van der Hoorn RAL. Ten prominent host proteases in plant-pathogen interactions. Int J Mol Sci. 2018;19:639. 29495279 10.3390/ijms19020639 PMC5855861

[49] Crosatti C , Rizza F, Badeck FW et al. Harden the chloroplast to protect the plant. Physiol Plant. 2013;147:55–63. 22938043 10.1111/j.1399-3054.2012.01689.x

[50] van der Hoorn RAL . Plant proteases: from phenotypes to molecular mechanisms. Annu Rev Plant Biol. 2008;59:191–223. 18257708 10.1146/annurev.arplant.59.032607.092835

[51] le Roux ML , Kunert KJ, van der Vyver C et al. Expression of a small ubiquitin-like modifier protease increases drought tolerance in wheat (Triticum aestivum L.). Front Plant Sci. 2019;10:266. 30906307 10.3389/fpls.2019.00266 PMC6418343

[52] Yao X , Xiong W, Ye TT et al. Overexpression of the aspartic protease ASPG1 gene confers drought avoidance in Arabidopsis. J Exp Bot. 2012;63:2579–93. 22268147 10.1093/jxb/err433 PMC3346222

[53] Chen HJ , Su CT, Lin CH et al. Expression of sweet potato cysteine protease SPCP2 altered developmental characteristics and stress responses in transgenic Arabidopsis plants. J Plant Physiol. 2010;167:838–47. 20129700 10.1016/j.jplph.2010.01.005

[54] Mishra RC , Richa, Grover A. Constitutive over-expression of rice ClpD1 protein enhances tolerance to salt and desiccation stresses in transgenic Arabidopsis plants. Plant Sci. 2016;250:69–78. 27457985 10.1016/j.plantsci.2016.06.004

[55] Jones JT , Mullet JE. A salt-inducible and dehydration-inducible pea gene, Cyp15a, encodes a Cell-Wall protein with sequence similarity to cysteine proteases. Plant Mol Biol. 1995;28:1055–65. 7548823 10.1007/BF00032666

[56] Hatsugai N , Kuroyanagi M, Yamada K et al. A plant vacuolar protease, VPE, mediates virus-induced hypersensitive cell death. Science. 2004;305:855–8. 15297671 10.1126/science.1099859

[57] Adam Z , Rudella A, van Wijk KJ. Recent advances in the study of Clp, FtsH and other proteases located in chloroplasts. Curr Opin Plant Biol. 2006;9:234–40. 16603408 10.1016/j.pbi.2006.03.010

[58] Grudkowska M , Zagdanska B. Multifunctional role of plant cysteine proteinases. Acta Biochim Pol. 2004;51:609–24. 15448724

[59] Muntz K . Protein dynamics and proteolysis in plant vacuoles. J Exp Bot. 2007;58:2391–407. 17545219 10.1093/jxb/erm089

[60] Tiwari LD , Mittal D, Mishra RC et al. Constitutive over-expression of rice chymotrypsin protease inhibitor gene OCPI2 results in enhanced growth, salinity and osmotic stress tolerance of the transgenic Arabidopsis plants. Plant Physiol Biochem. 2015;92:48–55. 25910649 10.1016/j.plaphy.2015.03.012

[61] Huang YM , Xiao BZ, Xiong LZ. Characterization of a stress responsive proteinase inhibitor gene with positive effect in improving drought resistance in rice. Planta. 2007;226:73–85. 17221232 10.1007/s00425-006-0469-8

[62] Srinivasan T , Kumar KRR, Kirti PB. Constitutive expression of a trypsin protease inhibitor confers multiple stress tolerance in transgenic tobacco. Plant Cell Physiol. 2009;50:541–53. 19179349 10.1093/pcp/pcp014

[63] Cui XD , Wang ZH, Li YY et al. Buckwheat trypsin inhibitor enters Hep G2 cells by clathrin-dependent endocytosis. Food Chem. 2013;141:2625–33. 23871004 10.1016/j.foodchem.2013.04.001

[64] Trusova SV , Golyshev SA, Chichkova NV et al. Sometimes they come back: endocytosis provides localization dynamics of a subtilase in cells committed to cell death. J Exp Bot. 2019;70:2003–7. 30668760 10.1093/jxb/erz014 PMC6460962

[65] Trusova SV , Teplova AD, Golyshev SA et al. Clathrin-mediated endocytosis delivers Proteolytically active Phytaspases into plant cells. Front Plant Sci. 2019;10:873. 31379892 10.3389/fpls.2019.00873 PMC6657458

[66] Nielsen E , Cheung AY, Ueda T. The regulatory RAB and ARF GTPases for vesicular trafficking. Plant Physiol. 2008;147:1516–26. 18678743 10.1104/pp.108.121798 PMC2492611

[67] Ueda T , Yamaguchi M, Uchimiya H et al. Ara6, a plant-unique novel type Rab GTPase, functions in the endocytic pathway of Arabidopsis thaliana. EMBO J. 2001;20:4730–41. 11532937 10.1093/emboj/20.17.4730 PMC125591

[68] Grosse-Holz FM , van der Hoorn RAL. Juggling jobs: roles and mechanisms of multifunctional protease inhibitors in plants. New Phytol. 2016;210:794–807. 26800491 10.1111/nph.13839

[69] Yang H , Li H, Rao LQ et al. Effects of exogenous ABA on antioxidant enzymes in detached citrus leaves treated by rapid freezing. Afr J Biotechnol. 2011;10:9779–85.

[70] Buege JA , Aust SD. Microsomal lipid peroxidation. Methods Enzymol. 1978;52:302–10. 672633 10.1016/s0076-6879(78)52032-6

[71] Yang H , Li H, Rao LQ et al. A method for isolation of DNA- binding proteins based on solubility of DNA- protein complexes. Protein Pept Lett. 2012;19:1071–5. 22512647 10.2174/092986612802762651

[72] Livak KJ , Schmittgen TD. Analysis of relative gene expression data using real-time quantitative PCR and the 2(T)(-Delta Delta C) method. Methods. 2001;25:402–8. 11846609 10.1006/meth.2001.1262

