# Supplementary material for: Protease inhibitor ASP enhances freezing tolerance by inhibiting protein degradation in kumquat
Source: Hortic Res. 2023 Feb 16;10(4):uhad023. doi: 10.1093/hr/uhad023 (PMC10541525; doi:10.1093/hr/uhad023)
Supplement: Web_Material_uhad023 [file web_material_uhad023.zip › Supplementary_Figure_S1-S4.pptx]

## Slide 1
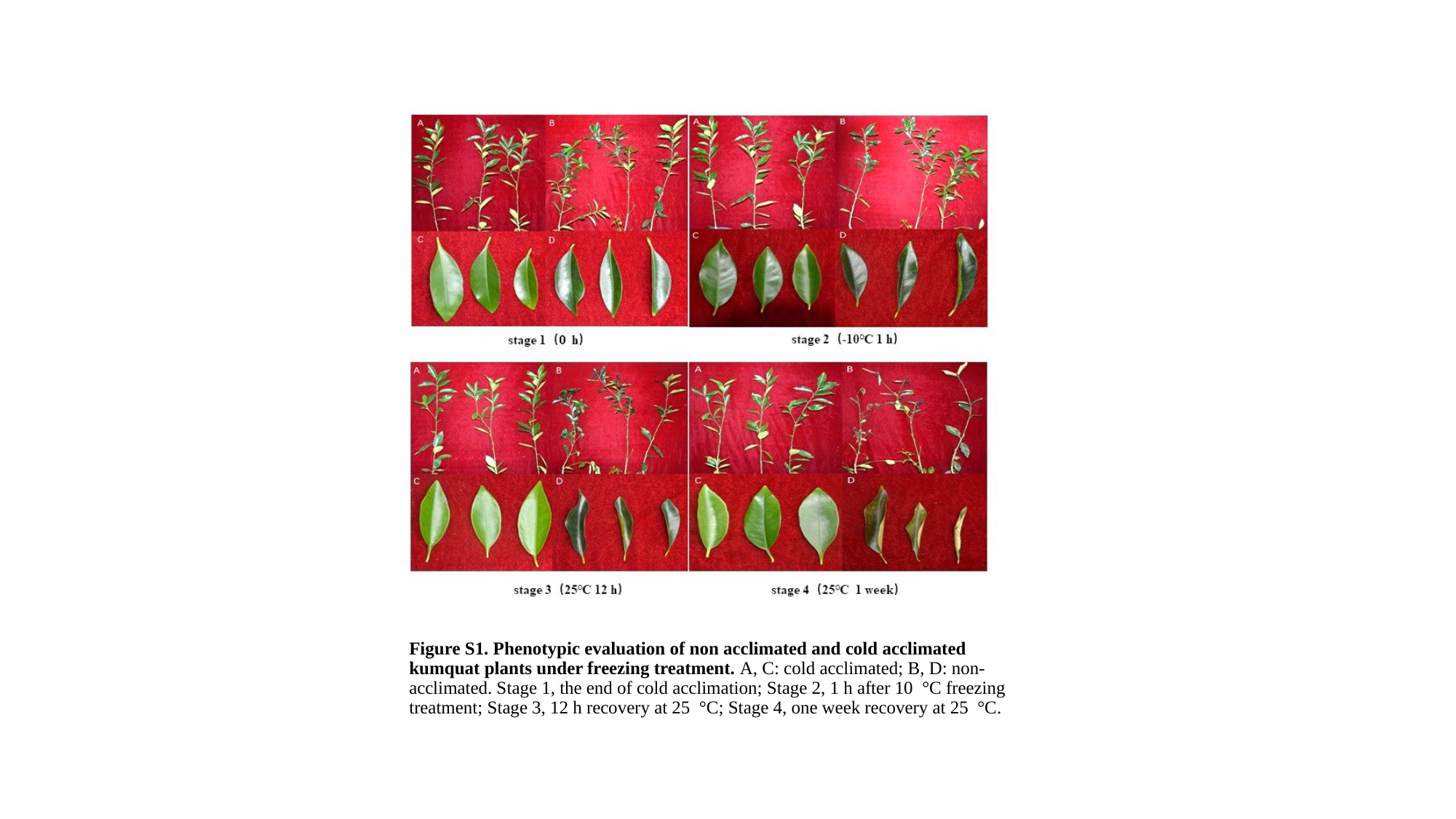

Figure S1. Phenotypic evaluation of non acclimated and cold acclimated kumquat plants under freezing treatment. A, C: cold acclimated; B, D: non-acclimated. Stage 1, the end of cold acclimation; Stage 2, 1 h after 10 °C freezing treatment; Stage 3, 12 h recovery at 25 °C; Stage 4, one week recovery at 25 °C.

## Slide 2
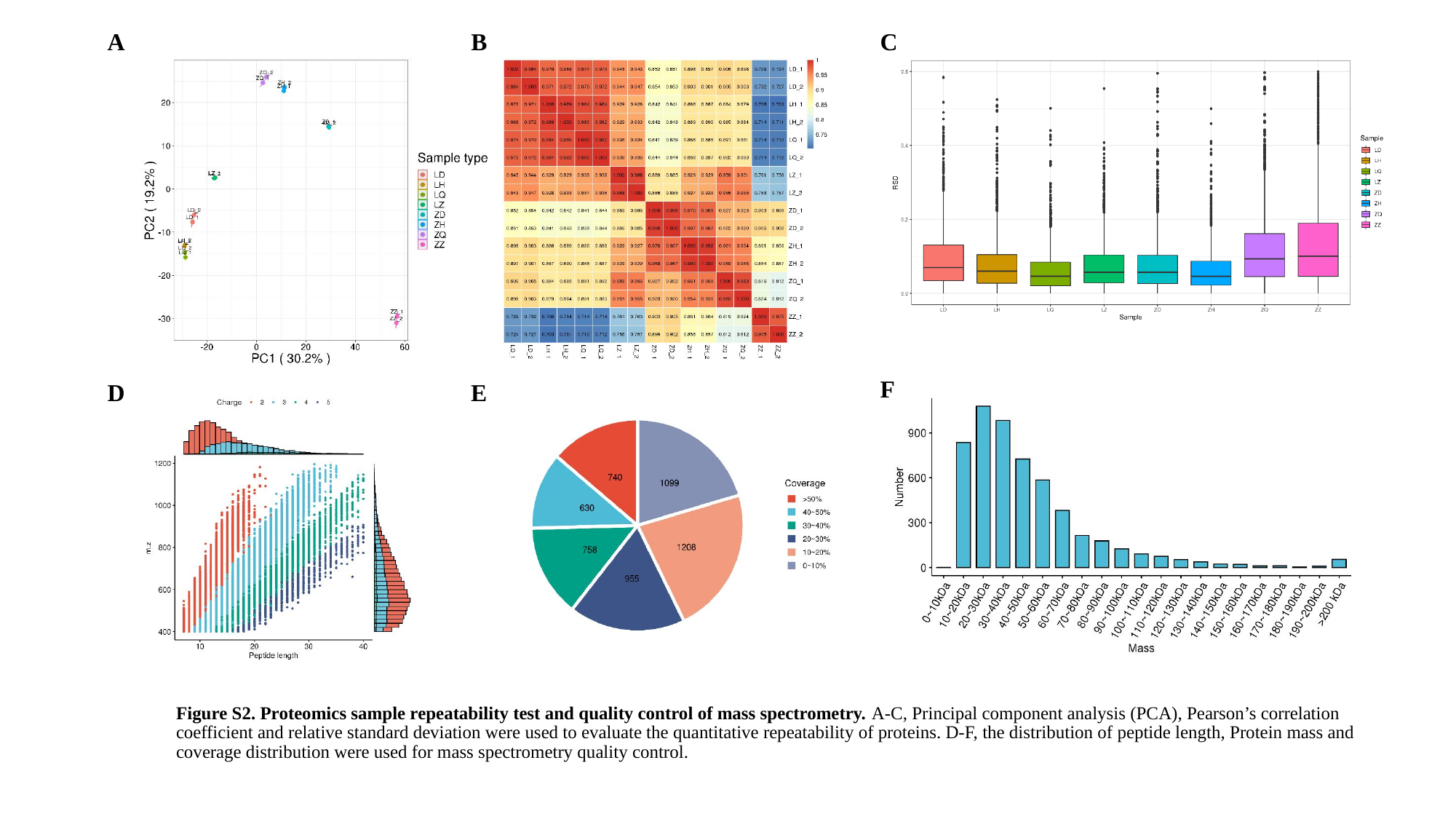

A
B
C
F
D
E
Figure S2. Proteomics sample repeatability test and quality control of mass spectrometry. A-C, Principal component analysis (PCA), Pearson’s correlation coefficient and relative standard deviation were used to evaluate the quantitative repeatability of proteins. D-F, the distribution of peptide length, Protein mass and coverage distribution were used for mass spectrometry quality control.

## Slide 3
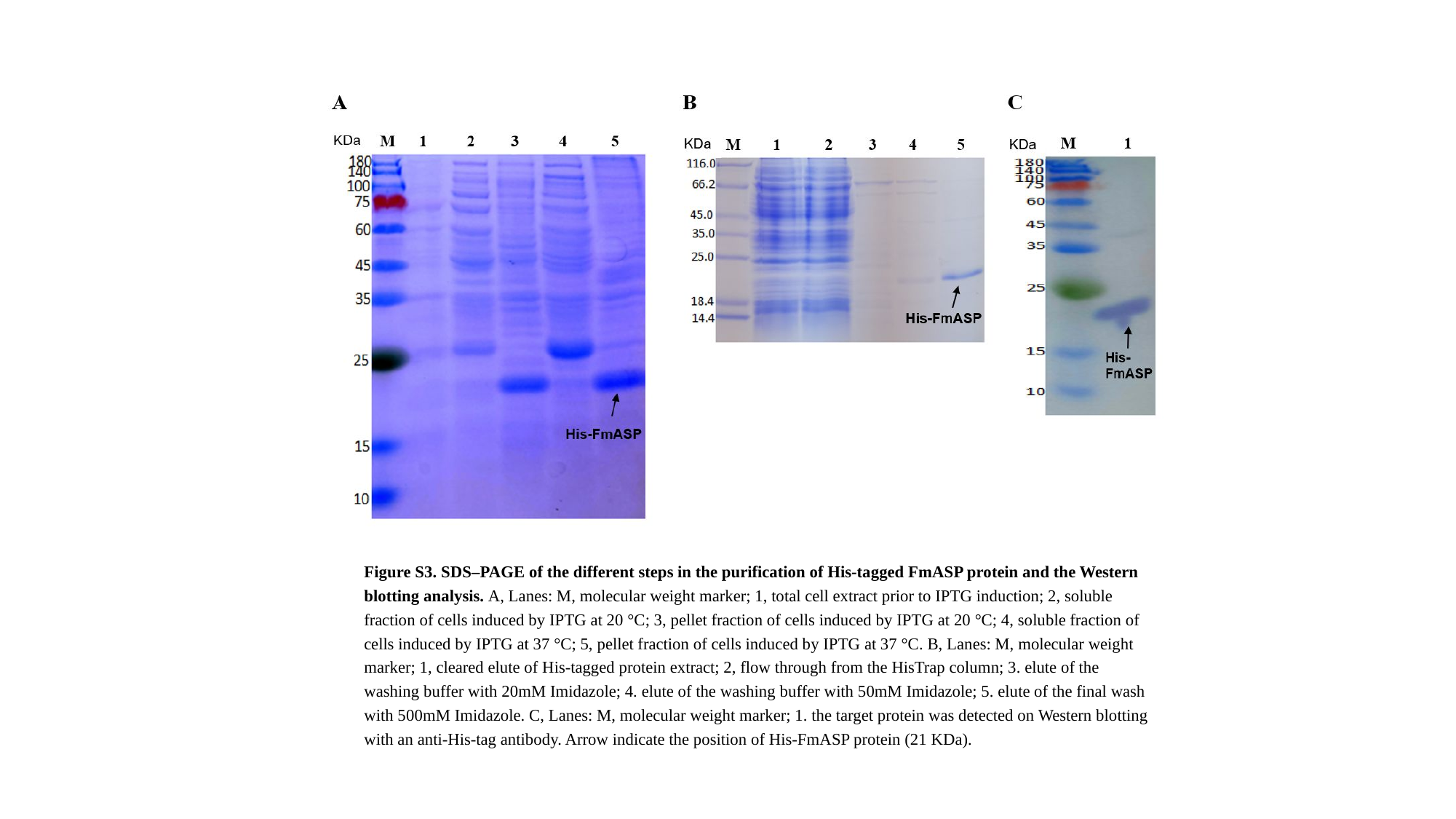

Figure S3. SDS–PAGE of the different steps in the purification of His-tagged FmASP protein and the Western blotting analysis. A, Lanes: M, molecular weight marker; 1, total cell extract prior to IPTG induction; 2, soluble fraction of cells induced by IPTG at 20 °C; 3, pellet fraction of cells induced by IPTG at 20 °C; 4, soluble fraction of cells induced by IPTG at 37 °C; 5, pellet fraction of cells induced by IPTG at 37 °C. B, Lanes: M, molecular weight marker; 1, cleared elute of His-tagged protein extract; 2, flow through from the HisTrap column; 3. elute of the washing buffer with 20mM Imidazole; 4. elute of the washing buffer with 50mM Imidazole; 5. elute of the final wash with 500mM Imidazole. C, Lanes: M, molecular weight marker; 1. the target protein was detected on Western blotting with an anti-His-tag antibody. Arrow indicate the position of His-FmASP protein (21 KDa).

## Slide 4
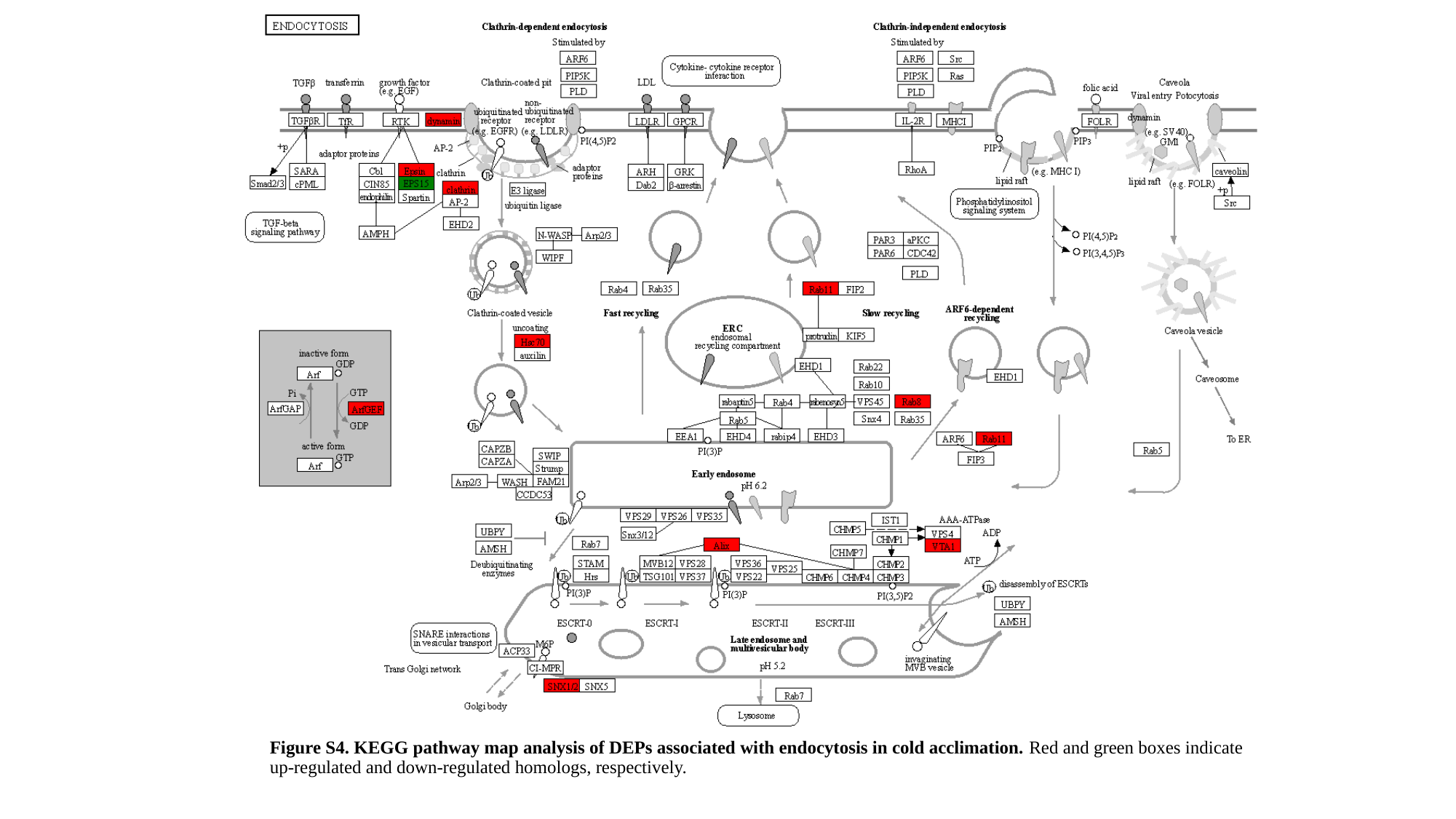

Figure S4. KEGG pathway map analysis of DEPs associated with endocytosis in cold acclimation. Red and green boxes indicate up-regulated and down-regulated homologs, respectively.
